# *In vitro *modeling of host-parasite interactions: the 'subgingival' biofilm challenge of primary human epithelial cells

**DOI:** 10.1186/1471-2180-9-280

**Published:** 2009-12-31

**Authors:** Bernhard Guggenheim, Rudolf Gmür, Johnah C Galicia, Panagiota G Stathopoulou, Manjunatha R Benakanakere, André Meier, Thomas Thurnheer, Denis F Kinane

**Affiliations:** 1 Institute for Oral Biology, Section for Oral Microbiology and General Immunology, University of Zürich, Plattenstrasse 11, CH-8032, Zürich, Switzerland; 2 Oral Health and Systemic Disease Research Group, University of Louisville School of Dentistry, 501 S Preston St., Louisville, KY 40222, USA; 3 Department of Pathology and Periodontology, School of Dental Medicine, University of Pennsylvania, 240 South 40th Street, Philadelphia, PA19104-6030, USA

## Abstract

**Background:**

Microbial biofilms are known to cause an increasing number of chronic inflammatory and infectious conditions. A classical example is chronic periodontal disease, a condition initiated by the subgingival dental plaque biofilm on gingival epithelial tissues. We describe here a new model that permits the examination of interactions between the bacterial biofilm and host cells in general. We use primary human gingival epithelial cells (HGEC) and an *in vitro *grown biofilm, comprising nine frequently studied and representative subgingival plaque bacteria.

**Results:**

We describe the growth of a mature 'subgingival' *in vitro *biofilm, its composition during development, its ability to adapt to aerobic conditions and how we expose *in vitro *a HGEC monolayer to this biofilm. Challenging the host derived HGEC with the biofilm invoked apoptosis in the epithelial cells, triggered release of pro-inflammatory cytokines and in parallel induced rapid degradation of the cytokines by biofilm-generated enzymes.

**Conclusion:**

We developed an experimental *in vitro *model to study processes taking place in the gingival crevice during the initiation of inflammation. The new model takes into account that the microbial challenge derives from a biofilm community and not from planktonically cultured bacterial strains. It will facilitate easily the introduction of additional host cells such as neutrophils for future biofilm:host cell challenge studies. Our methodology may generate particular interest, as it should be widely applicable to other biofilm-related chronic inflammatory diseases.

## Background

In most natural environments bacteria exist as highly structured dense surface attached aggregates designated as biofilms [1,2]. This applies also to bacteria colonizing the skin and human mucosa. Under certain conditions, biofilms may cause disease. Classical examples are gingivitis and chronic inflammatory periodontal disease. Dental plaque colonizing teeth initiates inflammation in the adjacent host gingival epithelium. The epithelial cells lining the crevice between the gum and the tooth are the first line of defense to the plaque bacteria [3]. Dental plaque has long been recognized as a complex polymicrobial biofilm [1,4-6]. The maturation of this biofilm involves a change in the microbiota from predominantly Gram-positive facultative anaerobes to Gram-negative anaerobic bacteria [7]. As the plaque accumulates, it induces inflammation in the adjacent host tissues and the biofilm over time extends under the gum, down the root surface, creating a niche favoring the growth of fastidious anaerobes, such as *Spirochaetes *and *Bacteriodetes *[5].

Until very recently, *in vitro *experiments to elucidate this host-parasite relationship, utilized human cell line cultures challenged with putative pathogenic periodontal bacteria that were invariably used in the planktonic state, that is as cell suspensions in growth media or buffered solutions. Evidence from such experiments is far disconnected from *in vivo *conditions and although useful as a first approach, they poorly reflect the challenge to host cells by multi-species biofilms. Biofilms are comprised of either mono-species or multi-species biocenoses [8], and their eradication is more difficult than for planktonic bacteria as they are highly resistant to antimicrobial agents and the host's immune response [9]. Pathogenic biofilms are often associated with chronic inflammatory diseases such as periodontitis, or chronic conditions that are difficult to treat such as the colonization of urinary catheters and endotracheal tubings [2,10-14]. Biofilm or co-culture studies composed of one to three bacteria have been reported in the past [15-17]; however, with hundreds of different bacteria present in the human mouth, a more extensive biofilm study model will better elucidate the cellular responses triggered by bacteria that usually colonize with other bacteria *in vivo*.

We developed an *in vitro *biofilm model mimicking subgingival plaque to challenge cultured primary gingival epithelial cells (HGEC) in order to assess interactions that may reflect more accurately the *in vivo *processes occurring in the gingival epithelium during the initiation of periodontal inflammation. In comparison to epithelial cell lines, HGECs are optimal, since the former have characteristics and receptors similar to fibroblasts and other cell types [18]. The bacterial species incorporated in the *in vitro *'subgingival' biofilm were chosen carefully based on the following criteria: 1) published reports that the species were frequently found and numerous in periodontal disease sites; and 2) that the species were cultivatable to permit enumeration and also measurable by fluorescent *in situ *hybridization (FISH) and if possible immunofluorescence (IF), in additional species-specific single-cell detection assays to ensure quality control. We thus compiled a list of nine microorganisms that, when incorporated in a biofilm, included widely accepted pathogenic microbiota [19-21]. Besides members of the gingival crevice plaque (e.g. *Actinomyces naeslundii, Streptococcus oralis, Veillonella dispar*) it comprises taxa detected most commonly in deep periodontal pockets and widely utilized in virulence experiments (e.g. *Porphyromonas gingivalis, Prevotella intermedia, Tannerella forsythia*) with planktonic cells.

We describe here the procedures to generate the complex 'subgingival' *in vitro *biofilms and the techniques used to challenge HGEC with such biofilms. We report on the kinetics and reproducibility of biofilm formation and on the effects of the biofilm on epithelial cells in terms of generating apoptosis, inducing pro-inflammatory primary and secondary cytokines, and triggering direct biofilm-mediated cytokine degradation.

## Results

### Characterization of biofilm composition

**Figure 1:**
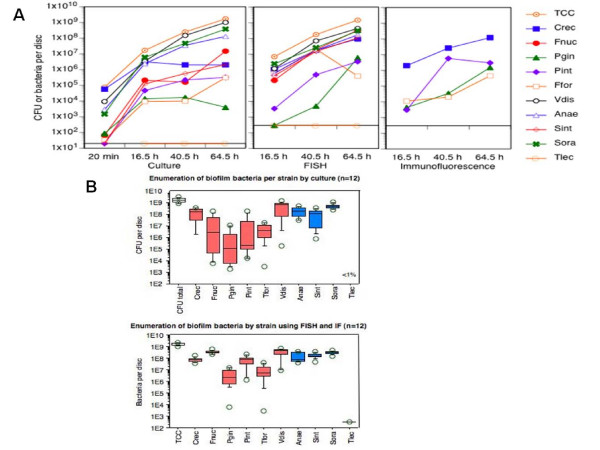
**Characterization of 9-species 'subgingival' in vitro biofilm model**. **(A) **Time flow of biofilm formation. Data are means from triplicate biofilms and gained comparatively by culture analysis (left panel), FISH with taxa-specific 16S rRNA probes (central panel), or indirect IF with species-specific mAb (right panel, only four taxa). **(B) **Total bacteria and all individual taxa of 64.5 h biofilms enumerated by culture (upper panel) and FISH/IF(lower panel), assessing four completely independent experiments, each performed with triplicate biofilms. Culture data are expressed as CFU per biofilm, FISH/IF data as number of bacteria per biofilm. Box colors indicate Gram-negative (red) or Gram-positive (blue) bacteria. Abbreviations: TCC, total cell count; Crec, *Campylobacter rectus*; Fnuc, *F. nucleatum subsp. vincentii*; Pgin, *Porphyromonas gingivalis*; Pint, *Prevotella intermedia*; Tfor, *Tannerella forsythia*; Vdis, *Veillonella dispar*; Anae, *Actinomyces naeslundii*; Sint, *Streptococcus intermedius*; Sora, *Streptococcus oralis*.

We generated *in vitro *'subgingival' biofilms containing nine different bacterial species representative of marginal and subgingival plaque. We determined the kinetics of biofilm formation using three independent bacteria detection and enumeration assays to quantitate all biofilm members (Fig. 1A). Scraped from the surface of the hydoxyapatite discs after only 20 min of anaerobic incubation to evaluate initial adherence, the bacteria showed large quantitative inter-species differences. *Campylobacter rectus *accounted for nearly 90% of the cells at this time point. *V. dispar, A. naeslundii*, and *S. oralis *were in 1-10% range, whereas the other five organisms were all below 0.1% of the total CFU. FISH and IF, as optical single cell identification techniques, were not sufficiently sensitive to reliably enumerate all nine species at 20 min.

Cell accumulation in the biofilm was fastest during the initial presence of the inoculum suspension (up to 16.5 h). Thereafter, the total cell number (TCC) as well as the cell numbers of the individual species continued to increase by approximately two log steps until the experiments were stopped at 64.5 h of biofilm formation. Culture and FISH analyses yielded very similar data for *A. naeslundii, S. oralis*, and *V. dispar*. The other more fastidious taxa were detected in somewhat higher numbers by FISH and IF (only four species studied by IF). Cell viability in 64.5 h biofilms was estimated microscopically by the in-/exclusion of fluorescent dyes and reached consistently 85%.

The reproducibility of biofilm formation was studied by performing four completely independent experiments, each in triplicate and at least one week apart from each other. When examined by optical single cell analysis(Fig. 1B, lower graph), little variation was observed between independent experiments for all but the two putative subgingival pathogens *P. gingivalis*, and *T. forsythia*, suggesting that these fastidious organisms had the most difficulties to establish in the biofilms. This interpretation is supported by the corresponding culture data (Fig. 1B, upper graph), where, together with *Fusobacterium nucleatum *and *P. intermedia*, these species showed the lowest level of colonization and the largest variation. *F. nucleatum *and *P. intermedia *colonized the biofilms consistently at high densities (Fig. 1B, lower graph) but were markedly underscored by culture due to their fastidious growth requirements. Changes of the nutritional conditions resulted in the formation of biofilms of quantitatively different composition (Table 1). In general, an increase of the serum concentration at the cost of the saliva concentration led to higher total CFU per biofilm and to equal or higher CFU of the individual species. *P. intermedia *was a notable exception by colonizing best in the medium with 30% mFUM, 60% saliva and only 10% serum which are the conditions used in the following for our co-culture experiments with HGEC.

**Table 1:** Composition of biofilms generated under different nutritional conditions.

Culture Conditions		Number of bacteria (± SD) present in biofilm per disc (cfu, ×10^6^)										
**0-16.5 h**	**16.5 - 64.5 h**	**N**	**Total**	**Crec**	**Fnuc**	**Pgin**	**Pint**	**Tfor**	**Vdis**	**Anae**	**Sint**	**Sora**

a	b	15	3450 (1780)	365 (151)	50 (38)	60 (48)	1.4 (1.2)	11 (14)	614 (601)	155 (133)	405 (311)	982 (488)

c	d	12	1770 (719)	171 (140)	36 (61)	2.3 (4.4)	30 (63)	6.8 (7.0)	541 (458)	220 (165)	122 (117)	520 (268)

a	e	12	2710 (1100)	256 (211)	26 (43)	105 (151)	1.7 (3.1)	3.0 (2.7)	931 (702)	103 (122)	219 (190)	686 (309)

a	f	3	710 (529)	1.4 (1.3)	0.01 (0.004)	7.3 (7.6)	0.08 (0.003)	47 (30)	142 (103)	23 (3)	61 (22)	293 (55)

Culture conditions: **a**, 35% saliva + 35% human serum + 30% FUM with 3% glucose, anaerob (10% H_2_, 5% CO_2_, 85% N_2_); **b**, 35% saliva + 35% human serum + 30% FUM with 3% glucose, aerob (5% O_2_, 5% CO_2_, 90% N_2_); **c**, 60% saliva + 10% human serum + 30% FUM with 3% glucose, anaerob (10% H_2_, 5% CO_2_, 85% N_2_); **d**, 60% saliva + 10% human serum + 30% FUM with 3% glucose, aerob (5% O_2_, 5% CO_2_, 90% N_2_); **e**, 70% human serum + 30% FUM with 3% glucose, aerob (5% O_2_, 5% CO_2_, 90% N_2_); **f**, 70% human serum + 30% 0.9% NaCl, aerob (5% O_2_, 5% CO_2_, 90% N_2_). Species abbreviations are as defined in the legend to Figure 1.

### Biofilm structure

**Figure 2:**
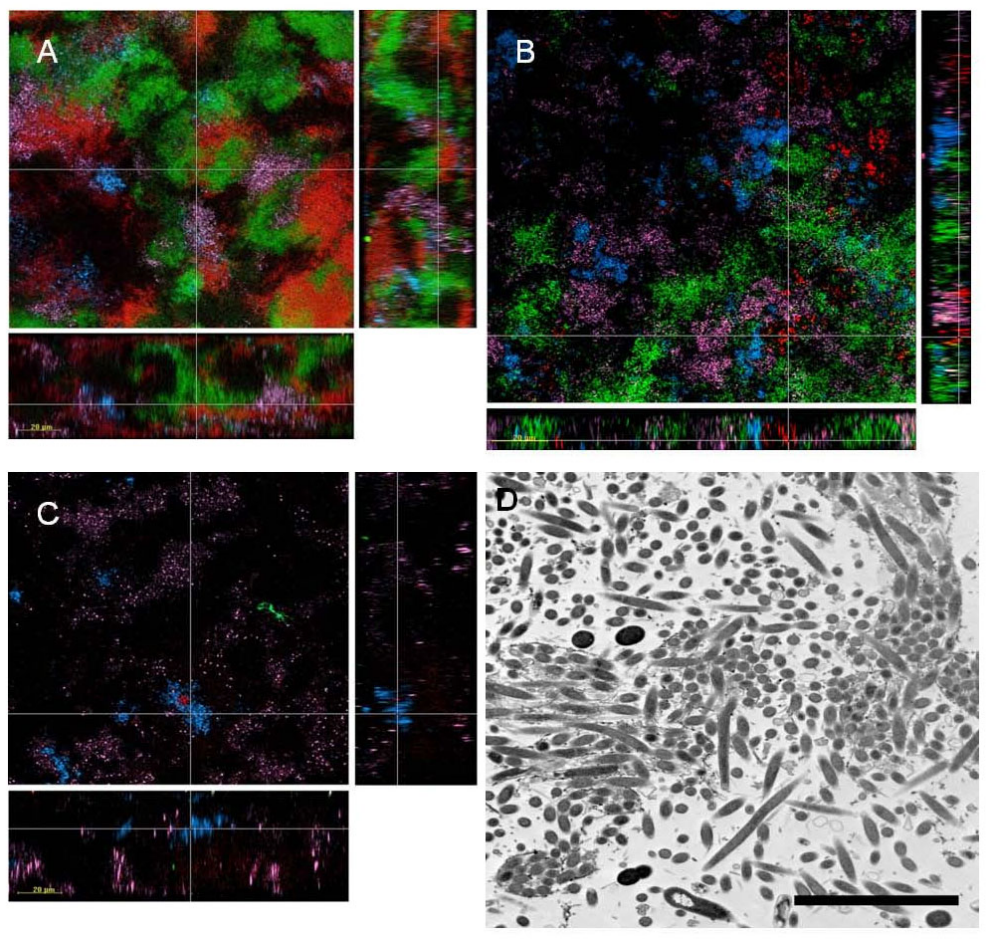
**Biofilm structure visualized by CLSM and TEM**. CLSM images of a 64.5 h 9-species biofilm stained by multiplex FISH for **(A) ***V. dispar *(purple; VEI217-ROX, 40% formamide), *C. rectus *(blue; Camp655-Cy5, 30% formamide), *F. nucleatum *(red; Fnuc133c-Cy3, 30% formamide), and *P. intermedia *(green; L-Pint649-2-FAM, 30% formamide), **(B) ***V. dispar *(purple; VEI217-ROX, 40% formamide), *A. naeslundii *(red; L-Act476-2-Cy3, 25% formamide), *S. intermedius *(green; L-Sco/int172-2-FAM, 25% formamide), and *S. oralis *(blue; MIT447-Cy5, 25% formamide), and **(C) ***V. dispar *(purple; VEI217-ROX, 40% formamide), *T. forsythia *(green; Tfor582-FAM, 40% formamide), *P. gingivalis *(red; L-Pgin1006-Cy3, 30% formamide), and *C. rectus *(blue; Camp655-Cy5, 30% formamide). Images are 1-*μ*m-thick transverse (large images), sagittal (right) and coronal (bottom) slices at the positions indicated by the fine lines. The length of the bars indicates 20 *μ*m. **(D) **TEM image of a 64.5 h multi-species biofilm demonstrating the predominance of varius cocci or very short rods (*S. oralis, S. intermedius, V. dispar, P. intermedia*) and of the fusiform *F. nucleatum *cells. Bar = 5 *μ*m.

Bacteria were stained by multiplex FISH and assessed by confocal laser scanning microscopy (CLSM). Representative images are shown in Fig. 2. 64.5 h biofilms had a thickness of 40 to 60 *μ*m with broad distribution of *S. intermedius*, *P. intermedia *and *F. nucleatum*. In contrast, *T. forsythia*, and *P. gingivalis *were restricted to microcolonies. *C. rectus *occurred dispersed, but here and there also in microcolonies. Images from transmission electron microscopy (TEM) show the predominance of various cocci and short rods (streptococci, *P. intermedia, V. dispar*) interspersed by prominent elongated fusiform cells (*F. nucleatum *subsp. *vincentii*) (Fig. 2D).

### The *in vitro *'subgingival' biofilm induces apoptosis in HGEC

**Figure 3:**
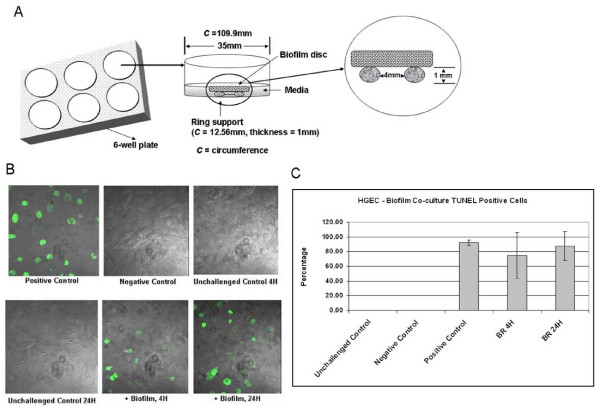
**Apoptosis of human cultured gingival epithelial cells exposed to the biofilm**. **(A) **Scheme of the co-culture design as used in both single cell culture dishes and 6-well plates. Biofilms were placed upside-down on a ring support that was layed onto the gingival epithelial cell layer. **(B) **TUNEL labeling of apoptotic HGEC exposed to the biofilm using confocal microscopy at ×600 magnification. Positive control - complete TUNEL assay of DNAse-treated HGEC; negative control - TUNEL label solution only on HGEC in plain KSFM; unchalleged controls - complete TUNEL assay of HGEC in plain KSFM without biofilm challenge; biofilm on ring - HGEC challenged for 4 h and 24 h with the biofilm on a ring support (see Fig. 3A). **(C) **Percentage of TUNEL positive apoptotic HGEC exposed to the biofilm, estimated using confocal microscopy. Unchalleged controls - complete TUNEL assay of HGEC in plain KSFM without biofilm challenge; negative control - TUNEL label solution only on HGEC in plain KSFM; positive control - complete TUNEL assay of DNAse-treated HGEC; biofilm on ring - HGEC challenged with the biofilm on a ring support (see Fig. 3A) for 4 and 24 h. Values represent the mean ± SD of at least 4 fields of vision from 2 assays.

HGEC in 6-well plates were challenged with biofilms attached to a hydroxyapatite (HA) disc for 4 and 24 h using the experimental setup described in Fig. 3A. Apoptosis was detected by the TUNEL assay. Biofilm-challenged HGEC exhibited signs of apoptosis early during challenge (Fig. 3B). After only 4 h approximately 75% of the cells showed blebbing and pyknotic nuclei and stained positive for TUNEL. At 24 h more than 85% of the HGEC were apoptotic (Fig. 3C).

### Biofilm-challenged HGEC elicit a cytokine response that is reduced over time

**Figure 4:**
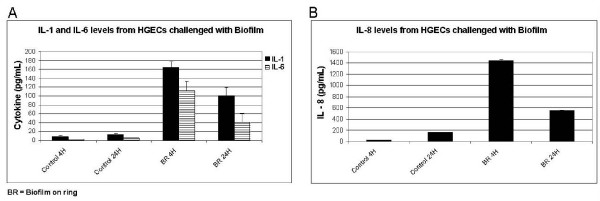
**Cytokine production in biofilm-challenged HGEC cultures**. **(A) **Supernatant IL-1 and IL-6 levels, and **(B) **IL-8 levels in challenged HGEC cultures and controls. Unchallenged cells were used as a negative control. Values represent the mean ± SD of triplicate ELISA assays performed after 4 and 24 h of co-culture.

HGEC were challenged with 'subgingival' biofilms for 4 and 24 h. At 4 h, the primary (IL-1β) and secondary cytokine (IL-6) and chemokine (IL-8) responses were significantly elevated (Fig. 4A &4B). At 24 h, however, the level of all cytokines had subsided significantly. To test our hypothesis that this decline of cytokine levels is due to biofilm-mediated degradation, supernatant from HGEC, pre-challenged with heat-killed planktonic *P. gingivalis *ATCC 33277 for establishing cytokine production, was incubated either with biofilms on HA discs, filtered supernatant from 64.5 h biofilm cultures, or with KSFM medium in which biofilms had been incubated for 24 h (filtered or unfiltered). Assays performed after 1 min, 30 min, 1, 2, and 4 h of exposure to the biofilm showed that IL-1β, IL-6 and IL-8 degradation began immediately and progressively increased to reach approximately 25% after 4 h (Table 2). When filtered biofilm culture supernatant or KSFM were tested, IL-6 and IL-8 degradation was not observed, while the rate of IL-1β degradation was significantly reduced up to 2 h but eventually reached levels similar to that induced by the biofilm at 4 h. This suggests that the presence of biofilm bacteria is necessary for IL-6 and IL-8 degradation, while IL-1β is more susceptible to degradation by a soluble component of the biofilm culture.

**Table 2:** Cytokine degradation in biofilm challenged HGEC cultures.

		Control			Biofilm			Supernatant			Media Filtered			Media Unfiltered		
		**Mean**	**SD**	***p*^+^**	**Mean**	**SD**	***p***	**Mean**	**SD**	***p***	**Mean**	**SD**	***p***	**Mean**	**SD**	***p***

**IL1β**	1 min	22.31	0.45	>0.05	17.31	0.78	**	19.94	1.05	>0.05	16.82	0.66	***	15.98	0.55	***
	.5 h	20.69	0.38	>0.05	14.73	0.37	***	20.08	0.35	>0.05	17.14	0.38	**	16.92	0.32	***
	1 h	18.44	0.69	>0.05	15.67	1.47	***	19.65	0.51	>0.05	16.40	0.31	***	14.39	0.21	***
	2 h	19.40	1.19	>0.05	15.43	0.73	***	18.80	0.99	>0.05	13.64	1.22	***	14.80	0.69	***
	4 h	17.82	2.43	>0.05	16.37	0.60	***	17.14	0.73	**	13.81	0.45	***	13.50	0.27	***

**IL6**	1 min	354.02	43.22	>0.05	309.97	2.77	>0.05	373.18	5.12	>0.05	315.48	0.92	>0.05	317.15	4.25	>0.05
	.5 h	383.02	2.32	>0.05	311.47	2.57	>0.05	372.69	0.33	>0.05	316.65	1.99	>0.05	284.25	0.81	>0.05
	1 h	391.68	4.46	>0.05	310.80	3.25	>0.05	361.35	1.66	>0.05	301.46	0.40	>0.05	288.43	1.16	>0.05
	2 h	394.68	3.57	>0.05	301.45	5.49	>0.05	353.85	1.80	>0.05	288.26	2.64	>0.05	290.77	0.77	>0.05
	4 h	323.98	31.60	>0.05	254.82	0.57	**	361.02	2.22	>0.05	294.61	0.75	>0.05	298.12	0.63	>0.05

**IL8**	1 min	1277.28	10.12	>0.05	1268.25	12.51	>0.05	1424.81	4.71	>0.05	1281.82	9.28	>0.05	1172.69	5.67	>0.05
	.5 h	1496.87	1.09	>0.05	1115.00	9.42	***	1406.66	2.64	>0.05	1142.27	34.56	>0.05	1164.16	0.73	***
	1 h	1432.74	6.52	>0.05	1063.69	12.17	***	1322.08	12.07	>0.05	1321.95	7.34	>0.05	1159.02	2.61	***
	2 h	1419.32	6.88	>0.05	1072.63	19.14	***	1360.15	1.78	>0.05	1325.05	8.30	>0.05	1115.37	9.80	***
	4 h	1354.82	9.10	>0.05	950.81	2.84	***	1362.09	16.79	>0.05	1237.07	1.60	>0.05	1090.25	20.08	***

*p *= *p *value

** *p *≤ 0.01

*** *p *≤ 0.001

+ Control *p *values are of its triplicates. Test *p *values are against controls.

## Discussion

We demonstrated that biofilms mimicking subgingival plaque can be established reproducibly *in vitro*. When exposed in co-culture to primary human epithelial cells, they induce cell apopotosis and production of pro-inflammatory primary and secondary cytokines. We further showed that the cytokines produced are immediately attacked by the biofilm. Thus, this co-culture model opposing a multi-species 'subgingival' biofilm to human oral epithelial cells mirrors two key aspects of the interactions taking place at the dental plaque:periodontal tissue interphase, namely the triggering of a strong pro-inflammatory host cell response and the simultaneous microbial subversion of host defense mechanisms. Our strategy to construct *in vitro *'subgingival' biofilms makes use of the well-characterized 'supragingival' Zürich biofilm model [22,23] made up of six species, which *in vivo *colonize preferentially at the tooth surface and at the gingival margin. By eliminating *Streptococcus mutans*/*sobinus *and *Candida albicans*, and addingthe putative periodontal pathogens *C. rectus, P. gingivalis, P. intermedia, and T. forsythia*, we assembled a consortium of microorganisms that much more mirrors subgingival plaque. *Streptococcus intermedius *completes the biofilm consortium, being a subgingival species also known for its association with dental implant infections, pyogenic infections, endocarditis, and various types of abscesses [24-26]. A second strategic aim was to move our *in vitro *'subgingival' biofilms from strictly anaerobic to aerobic atmospheric conditions that match those used to propagate mammalian cells. If successful, this would be a major advance towards combining two *in vitro *model systems that basically seem to exclude each other. Our data show that biofilm adaptation to 5% O_2 _is feasible. They further show that the colonization reproducibility is not yet completely satisfactory as far as the most fastidious and most oxygen- sensitive species *P. gingivalis*, and *T. forsythia *are concerned (Fig. 1 and Table 1). This indicates that oxygen consumption by the microbial consortium - creating a low reduction potential - can be somewhat variable among parallel biofilms. Preliminary evidence suggests that later exposure of biofilms to oxygen (e.g. at 44.5 h) does not improve the growth of the subgingival anaerobes (with the possible exception of *T. forsythia*) and does not significantly reduce their viability either. The duration of the exposure to aerobic conditions had remarkably little effect on the ratios between the bacteria within the community (data not shown).

We used FISH, and in part indirect IF, in addition to quantitative culture for monitoring biofilm composition. Our data demonstrate the importance of using one or two complementary techniques, as any single technique may fail under particular circumstances. Examples in this study are the consistently reliable enumeration of *F. nucleatum *subsp. *vincentii *by FISH but not by culture due to difficulties in optimizing the *F. nucleatum *culture medium (a strain-specific phenomenon). CLSM of biofilms stained simultaneously for multiple bacterial species by multiplex FISH revealed a remarkable biofilm thickness of approximately 50 *μ*m and provided preliminary evidence for microcolony formation by several biofilm organisms which is reminiscent of microcolonies found characteristically in subgingival plaques from deep progressed periodontal pockets. Primary HGEC challenged with such 'subgingival' biofilms were able to induce a cytokine response after 4 h of challenge (Fig. 4A &4B) and although this is not strictly comparable because we did not perform comprehensive side by side comparisons, the cytokine response was higher than the previously reported cytokine response elicited by single planktonic bacterial species [27,28]. However, when the exposure time was prolonged to 24 h, the level of all cytokines had diminished significantly. We tested the hypothesis that the cytokine levels are reduced due to extracellular biofilm-mediated degradation. In fact, IL-6, and IL-8 degradation began immediately and progressively increased to reach approximately 25% after 4 h of exposure, but only in the presence of intact biofilm. This suggests that the presence of biofilm bacteria is necessary for IL-6 and IL-8 degradation, while IL-1β was found to be more susceptible to degradation by a soluble component in the biofilm culture medium. Biofilm-induced cytokine degradation could be, at least in part, due to the gingipain activity of *P. gingivalis *or a protease of *T. forsythia *with similar trypsin-like activity. Previous studies have shown that *P. gingivalis *can induce cytokine degradation [28-30] that is mainly lysine-gingipain-dependent. The lower rate of the biofilm-induced cytokine degradation observed in the present study compared to that induced by *P. gingivalis *monocultures [28] is possibly dose-related. Plaque mediated cytokine degradation is a potential mechanism to explain the reduced levels of pro-inflammatory cytokines (IL-6) and chemokines (IL-8) found in the GCF of patients with periodontitis [31-35], as well as the GCF from sites with active periodontal disease or unresolved defects following treatment [33,36]. In addition to cytokine production, primary HGEC challenged *in vitro *with a 'subgingival' biofilm exhibited apoptosis as evidenced by DNA fragmentation, the hallmark of apoptosis. This is a potential mechanism to explain the apoptosis that is observed in the gingiva at sites of chronic bacteria-induced inflammation [37,38], particularly among the superficial cells of the junctional epithelium [38] and the fibroblasts and leucocytes of the connective tissue [37,38]. The high proportions of HGEC undergoing apoptosis in the described co-culture system, as well as the short exposure time that is necessary for the initiation of the apoptotic process, suggest that the *in vitro *'subgingival' biofilm is a highly pathogenic entity, and possibly more pathogenic than single bacterial strains.

## Conclusion

Our approach allowed us to directy link primary human gingival epithelial cells, being an integral part of the oral innate immune system, to an artificial, *in vitro *propagated 'subgingival' biofilm, and elicit various cell responses ranging from cytokine production to apoptosis. Our data indicate that, compared to responses triggered by planktonic individual species, the bacteria organized in an *in vitro *'subgingival' biofilm express even more damaging virulence factors neutralizing the host cells' pro-inflammatory defense. As neither the culturing of host defense cells nor the assembly of artificial biofilms is restricted to oral tissues and bacteria, the same strategy of challenging cultured host cells with *in vitro *propagated bacterial biofilms may be of general interest and could be applied to study other elusive chronic inflammatory diseases.

## Methods

### In vitro biofilm generation

Technically, the 10-species biofilms used in this study are an advancement of the six-species model described previously [23,39]. The following 10 strains were used: *C. rectus *OMZ 697, *F. nucleatum *subsp. *vincentii *KP-F2 (OMZ 596), *P. gingivalis *ATCC 33277^T ^(OMZ 925), *P. intermedia *ATCC 25611^T ^(OMZ 278), *T. forsythia *OMZ 1047, *T. lecithinolyticum *ATCC 700332^T ^(OMZ 684), *V. dispar *ATCC 17748^T ^(OMZ 493), *A. naeslundii *OMZ 745, *S. intermedius *ATCC 27335 (OMZ 512), and *S. oralis *SK 248 (OMZ 607). Biofilms were grown in 24-well polystyrene cell culture plates on sintered pellicle-coated HA discs (10.6 mm Ø) [40]. To initiate biofilm formation discs were covered for the first 16.5 h with 1.6 ml of growth medium consisting of 60% saliva, 10% human serum (pool from three donors), 30% mFUM (modified fluid universal medium) [41,42] and 200 *μ*l of a bacterial cell suspension prepared from equal volumes and densities of each strain. After 16.5 h of anaerobic incubation at 37°C, the inoculum suspension was removed by "dip-washing" [39] the discs, which then were transferred into wells with fresh medium (60% saliva, 10% human serum, 30% mFUM) and incubated in an oxygen-containing atmosphere (90% N_2_, 5% CO_2_, 5% O_2_) for further 48 h. During this time-period discs were dip-washed after 20.5, 24.5, 40.5, 44.5 and 48.5 h and given fresh medium after 40.5 h. After 64.5 h of incubation, the discs were vigorously vortexed for 1 min in 0.9% NaCl to harvest the biofilms or intact biofilm-discs were frozen at -80°C for further use. This cultivation procedure (which corresponds to the culture conditions c/d in Table 1) was selected for use with all biofilm:HGEC co-culture experiments described herein after broad evaluation of other nutritional conditions. Options tested included richer conditions during the first 16.5 h (35% saliva and 35% human serum along with 30% mFUM) and several variations of the saliva, serum and mFUM concentration during the period from 16.5 to 64.5 h (Table 1). Further pre-testing to evaluate the effect of biofilm exposure to an oxygen-containing atmosphere and to the co-incubation with HGECusing KSFM were also investigated; although variations were observed, the number of anaerobic bacteria that survived after exposure to an aerobic environment was comparable to the pre-exposure levels (data not shown).

### Analysis of biofilm composition

Suspensions of harvested bacteria were evaluated for cell viability using the LIVE/DEAD BacLight Bacterial Viability assay (Molecular Probes) as described [42].

#### Culture

Serial dilutions of suspended biofilm bacteria were prepared in 0.9% NaCl and 50 *μ*l aliquots were plated on Columbia blood agar supplemented with 5% whole human blood (to estimate total CFU, *A. naeslundii, C. rectus, S. intermedius, V. dispar*) and phosphomycin (*P. gingivalis, P. intermedia*), on mitis salivarius agar (*S. oralis*), on fastidious anaerobe agar with erythromycin, vancomycin, and norfloxacin (*F. nucleatum*) [42], and on modified OMIZ-W68 agar [43] supplemented with lactose, caseinoglycomacropeptide, *N*-acetylmuramic acid, and *N*-acetylglucosamine to detect *T. forsythia*. With the exception of mitis-salivarius-agar plates (10% CO_2_) plates were incubated anaerobically at 37°C for 72 h. Species identification was achieved by observation of colony morphology in conjunction with microscopic and FISH examination of cells from selected colonies. Data were scored for each species as CFU per biofilm. *T. lecithiolyticum *was estimated by dark field microscopy and FISH.

#### FISH and IF

Suspensions of biofilm bacteria were spotted and fixed directly on 24-well slides and processed for FISH exactly as described [44]. The sequences of the employed custom-synthesized probes(Microsynth) are listed in Table 3. *C. rectus, P. gingivalis, P. intermedia*, and *T. forsythia *were stained by IF with monoclonal antibodies (mAb) 212WR2 [45], 61BG1.3 [46], 37BI6.1 [41], and 103BF1.1 [45], respectively, using a sandwich assay [44]. FISH and IF stained fluorescent bacteria were scored in multiple randomly selected viewing fields using the previously described equipment and counting procedure [44] resulting in a lower detection limit of approximately 3 × 10^3 ^bacteria ml^-1^.

**Table 3:** 16S rRNA targeted DNA probe sequences, target sites and target taxa

DNA Probe	Sequence (5' to 3')^b^	Site	% Forma- mide	Target	Reference
EUB338	GCTGCCTCCCGTAGGAGT	338-55	30-50	All biofilm members	[52]
L-Act476-2	ATCCAGC**T**ACC**G**TCAACC	476-93	25-40	*A. naeslundii*	This study
CAMP655	CATCTGCCTCTCCCTYAC	655-72	30	*C. rectus*	This study
Fnuc133c	GTTGTCCCTANCTGTGAGGC	133-52	30-40	*F. nucleatum*	This study
L-Pgin1006-2	GTTTTCACCATCM**G**TCA**T**C	1006-24	30	*P. gingivalis*	This study
L-Pint649-2	CGTTGCGTGCAC**T**CAA**G**TC	649-67	30-50	*P. intermedia*	This study
L-MIT446-2	ACACYCGTTCTTCTCTTACAA	446-66	25-50	*S. oralis*	This study
MIT447	CACYCGTTCTTCTCTTACA	447-65	25	*S. oralis*	[47]
L-Scoint172-2	CAGTAAATGTTCT**T**ATGC**G**GTA	172-91	25-40	*S. intermedius*	This study
Tfor127	CTCTGTTGCGGGCAGGTTAC	127-46	30-40	*T. forsythia*	[44]
Tfor582	GCGGACTTAACAGCCCACCT	582-601	30-40	*T. forsythia*	[44]
L-Tlema738-2	GCGTCAATTATC**T**GCC**G**G	738-55	30	*T. lecithinolythicum*	This study
VEI217	AATCCCCTCCTTCAGTGA	217-34	25-50	*V. dispar*	[47]

^a ^Probes were labeled at the 5'-end with Cy3, Cy5, 6-carboxyfluorescein (FAM), or 6-carboxy-X-rhodamine (ROX). The designations of all probes containing locked-nucleic-acid (LNA) substitutions start with L-. ^b ^Bold printed characters indicate LNA substitutions [53]. LNA incorporated DNA probes (LNA/DNA probes) have been described to improve significantly FISH fluorescence intensity in comparison to conventional DNA probes with the same sequence [53]. Our data fully confirm these findings. Applied in FISH at 10-20% higher formamide concentration than the corresponding conventional DNA probe with dental plaque samples, we noticed no loss of specificity for any of the new LNA/DNA probes (data not shown).

### CLSM and TEM analysis of biofilm structure

For CLSM intact biofilms were prepared first for multiplex FISH [45]. Lysozyme treatment (1 min) was done only if the ensuing FISH included staining Gram-positive biofilm species. Following pre-hybridization (15 min, 46°C), the FISH procedure was executed two to three times per biofilm, always starting with the probe(s) requiring the highest formamide concentration [47]. After the last FISH cycle excess saline was gently aspirated from the discs without touching the biofilms. They were embedded upside-down in 20 *μ*l of Mowiol [48] and stored at room temperature in the dark for at least 6 h prior to microscopic examination. Stained biofilms were examined by CLSM at randomly selected positions [39] using a 100 × (numeric aperture 1.4) oil immersion objective, and filters set to 500-540 nm for detection of 6-carboxyfluorescein (FAM), 540-580 nm for Cy3, 590-630 nm for 5-carboxy-X-rhodamine (ROX) and 640-700 nm for Cy5. Image acquisition was done in 8-line average mode and the data were processed as described [39]. To perform TEM biofilms were harvested at 64.5 h, fixed for 1 h in 2.5% glutaraldehyde, washed, and treated for 2 h with 1% osmium tetraoxide, all in cacodylate buffer, pH 7.4. Dehydration was done in a graded series of ethanol and then propyleneoxide before embedding with Epon in flat polystyrene wells. The hardened, embedded biofilms were cut into wedge-shaped pieces and treated with 10% EDTA for 3 days thus removing the HA. Ultra-thin sections were contrasted with saturated, acidified uranyl acetate and lead citrate for 3 min each. Sections were viewed on a Philips EM400T transmission electron microscope at 60 kV.

### Gingival cell isolation and culture

Gingival tissue biopsies were obtained with informed consent from periodontally healthy patients undergoing crown-lengthening procedures at the University of Louisville School of Dentistry, Graduate Periodontics Clinic, according to an IRB approval. The gingiva was treated with 0.025% trypsin and 0.01% EDTA overnight at 4°C and HGEC were isolated as previously described [49]. The authenticity of the gingival epithelial cells was confirmed by immunohistochemistry with mAb against human pankeratin (Dako) and histologically by cell morphology. The HGEC were seeded in 60-mm plastic tissue culture plates coated with type-I collagen (BD Biocoat) and incubated in 5% CO_2 _at 37°C using KSFM medium (Invitrogen) containing 10 *μ*g ml^-1 ^of insulin, 5 *μ*g ml^-1 ^of transferrin, 10 *μ*M of 2-mercaptoethanol, 10 *μ*M of 2-aminoethanol, 10 mM of sodium selenite, 50 *μ*g ml^-1 ^of bovine pituitary extract, 100 units ml^-1 ^of penicillin/streptomycin and 50 ng ml^-1 ^of fungizone (complete medium). When the cells reached sub-confluence, they were harvested and sub-cultured as described [50].

### HGEC:biofilm challenge

HGEC cultures at the fourth passage were harvested and seeded at a density of 0.5 × 10^5 ^cells per well in a 6-well culture plate coated with type-I collagen, and maintained in 2 ml of complete medium. The HGEC used in this study was chosen on the basis of it having a 'median' responsiveness, sufficient quantity of cells for testing and as our previous study showed consistent immunological trends among different cultures [27]. Biofilms on HA discs were defrosted and carefully placed for 24 h ("revived") in fresh biofilm-medium (60% saliva, 10% human serum, 30% mFUM) or plain KSFM (for cytokine degradation experiments; as explained later) in an oxygen-containing atmosphere (90% N_2_, 5% CO_2_, 5% O_2_). When the HGEC reached confluence (approximately 10^6 ^cells per well), the HGEC were washed twice with fresh medium and then challenged for 4 or 24 h in antibiotic-free medium at 37°C in 5% CO_2 _with one biofilm carrying HA disc per well that had just been dipped three times in sterile normal saline solution. The co-culture set-up is illustrated in Fig. 3A. Discs were placed on the ring support (tip of a sterile plastic inoculation loop; Copan) with the biofilm towards the HGEC layer. A one millimeter distance between the biofilm and the HGEC was maintained to allow fluid flow [51]. Neither the ring nor the HA disc triggered any of the HGEC responses assayed in this study. The effect of freezing, thawing and reviving of biofilms has been carefully assessed (data not shown). Results showed only minor differences in CFU/disc between fresh biofilms and biofilms that had been frozen, shipped, thawed and revived by incubating anaerobically in biofilm medium for 24 h (data not shown).

### Cytokine production and degradation assay

IL-1β, IL-6 and IL-8 were measured by ELISA using the OptEIA kit (BD Biosciences) according to the manufacturer's instructions. The absorbance was read at 450 nm. *P. gingivalis *strains at low passage were grown anaerobically in GAM media (Nissui Pharmaceutical) for 2 days. After cultivation, the bacteria were harvested by centrifugation, washed in PBS (pH 7.4) and then heat-inactivated for 1 h at 60°C. Culture supernatants produced after a 24 h incubation of HGEC with heat-inactivated planktonic *P. gingivalis *(MOI:100) were incubated at 37°C with: (i) biofilm attached to HA discs (Biofilm), (ii) supernatant from the biofilm culture (64.5 h) in the specific mFUM media (Supernatant), (iii) supernatant from a 24 h "revival" incubation of biofilm discs in plain KSFM either filtered or unfiltered (media filtered and media unfiltered). The reaction was stopped at 1 min, 30 min, 1 h, 2 h and 4 h by placing the samples at -80°C until ELISA. All data are expressed as the mean ± SD of three experiments done in triplicate. Statistical analyses were performed by one-way analysis of variance (ANOVA) using the InStat program (GraphPad, San Diego, CA) with Bonferroni correction. Statistical differences were considered significant at the *p *< 0.05 level.

### TUNEL assay

Direct TUNEL (Terminal deoxynucleotidyl Transferase Fluorescein-dUTP Nick End Labeling) assay was performed using a commercially available kit (Cat No 11-684795001, Roche Applied Science). Untreated cells were used as a negative control and cells treated with DNase 1000 U/ml were used as a positive control. The assay was performed according to the manufacturer's instructions. Briefly, the HGEC were washed three times with PBS, fixed with 4% paraformaldehyde(pH 7.4) for 30 min at room temperature, washed twice, and then permeabilized with 0.1% Triton X (Sigma-Aldrich) for 3 minutes on ice. After two washes, the cells were incubated with the TUNEL reaction mixture for 60 min at 37°C and then washed three times before analysis by CLSM (FluoView 500, Olympus). The percentage of apoptotic cells was determined by counting TUNEL-positive cells, cells exhibiting pyknosis and blebbing, and all cells present in four representative fields of vision (× 600 magnification) from each of two cultures with which TUNEL assays were performed.

## Abbreviations

CLSM: confocal laser scanning microscopy; FAM: 6-carboxyflurescein; FISH: fluorescent in situ hybridization; HA: hydroxy apatite; HGEC: human gingival epithelial cells; IF: immunofluorescence; LNA: locked-nucleic-acid; mAb: monoclonal antibodies; mFUM: modified fluid universal medium; MOI: multiplicity of infection; ROX: 5-carboxy-X-rhodamine; TCC: total cell number; TEM: transmission electron microscopy.

## Authors' contributions

BG and DFK conceived and designed the study and participated in analyzing the data and drafting the manuscript. AM developed the biofilms and made the microbiological culture analyses. RG carried out FISH and IF analyses, participated in analyzing the data and drafted the manuscript. TT carried out the CLSM experiments. JCG carried out the co-culture experiments, performed the apoptosis tests and participated in drafting the manuscript. PGS participated in carrying-out the co-culture experiments, cytokine analyses and in drafting the manuscript. MB carried out the cytokine and data analyses. All authors read and approved the final manuscript. The authors declare no conflict of interest.
